# Neurocognitive biases from the lab to real life

**DOI:** 10.1038/s42003-023-04544-4

**Published:** 2023-02-08

**Authors:** Henri Vandendriessche, Stefano Palminteri

**Affiliations:** Institut national de la santé et de la recherche médicale (INSERM) & École normale supérieure (ENS), Paris, France

## Abstract

Behavioral results suggest that learning by trial-and-error (i.e., reinforcement learning) relies on a teaching signal, the prediction error, which quantifies the difference between the obtained and the expected reward. Evidence suggests that distinct cortico-striatal circuits are recruited to encode better-than-expected (positive prediction error) and worst-than-expected (negative prediction error) outcomes. A recent study by Villano et al.^1^ provides evidence for differential networks that underlie learning from positive and negative prediction errors in humans using real-life behavioral data. More specifically, they found that university students are more likely to update beliefs concerning grade expectations following positive rather than negative prediction errors.

Virtually all animals use past rewards and punishments to modify their future course of actions (a process referred to as reinforcement learning). Behavioral and computational theories suggest that reinforcement learning relies on a teaching signal, the prediction error (PE), which quantifies the difference between the obtained and the expected reward. PE minimization across trials and repetitions enables (animals and humans alike) to efficiently learn and adapt their behavior accordingly.

**Figure:**
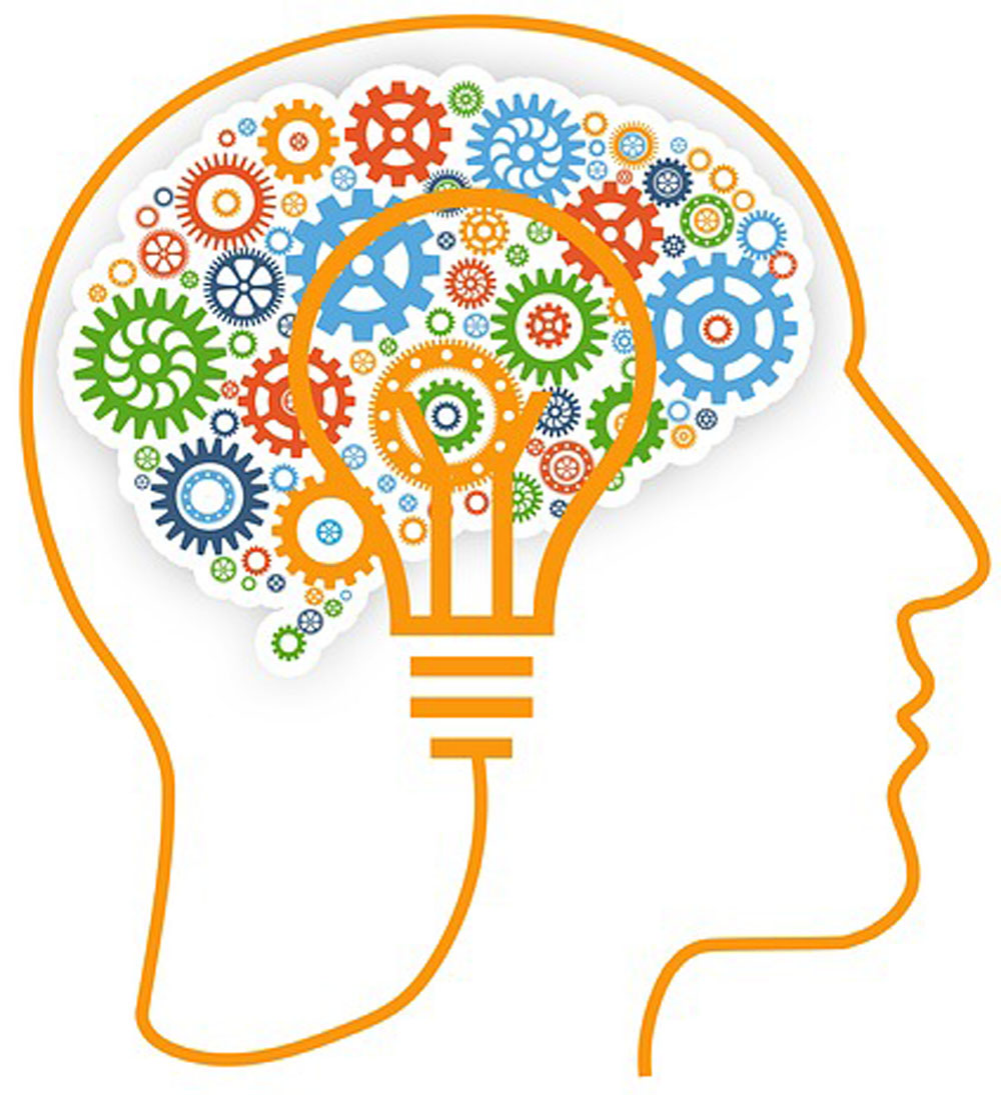
pixabay

Neurophysiological studies across rodents, monkeys and humans collectively suggest that reinforcement learning processes are largely underpinned by dopamine-mediated neural plasticity of the cortical-subcortical connections and that positive and negative PEs are encoded across dissociable circuits that recruit different cortical and subcortical structures of the brain^2^. A direct implication of these neurobiological observations is that learning from positive and negative PEs could be independently modulated at the behavioral level^3^.

Until recently, however, prior studies were carried out in relatively constrained and “aseptic” experimental settings. Most of the tasks employed implemented quite artificial learning scenarios, which potentially casts doubt on the ecological and real life validity of these findings.

A recent study from Villano et al.^1^ has overcome this limitation by investigating how humans integrate positive and negative PEs when updating their belief concerning consequential real life outcomes. More specifically, in a large scale study, they asked 625 university students to predict their grades during four consecutive semesters.

At the macroscopic level, participants learned to modify their expectations concerning future grades in a manner compatible with prediction error minimization. However, a more fine-grained analysis showed signs that could be considered as an “optimistic” bias: on average participants learned more following positive as opposed to negative PEs. The results support the idea of distinguishable neural networks for positive and negative prediction errors by showing an optimistic bias manifesting at the behavioral level.

The authors also report that participants who presented symptoms consistent with anxiety and depression, also showed a reduction in optimistic learning bias. This finding is consistent with prior evidence showing similar learning rate modifications in depression and anxiety^4^.

Altogether, this study provides evidence for prediction error-based learning outside of the laboratory, in real life scenarios. It also provides evidence for the fact that learning from positive and negative prediction errors are functionally dissociable. Both findings are consistent with decades-long neurobiological investigations of the computational mechanisms underlying reinforcement learning.
